# Overcoming Self-Incompatibility in Diploid Potato Using CRISPR-Cas9

**DOI:** 10.3389/fpls.2019.00376

**Published:** 2019-04-02

**Authors:** Felix Enciso-Rodriguez, Norma C. Manrique-Carpintero, Satya Swathi Nadakuduti, C. Robin Buell, Daniel Zarka, David Douches

**Affiliations:** ^1^Department of Plant, Soil and Microbial Sciences, Michigan State University, East Lansing, MI, United States; ^2^Department of Plant Biology, Michigan State University, East Lansing, MI, United States; ^3^Plant Resilience Institute, Michigan State University, East Lansing, MI, United States; ^4^AgBioResearch, Michigan State University, East Lansing, MI, United States

**Keywords:** self-incompatibility, diploid potato, *S-RNase*, CRISPR-Cas9, gene editing

## Abstract

Potato breeding can be redirected to a diploid inbred/F1 hybrid variety breeding strategy if self-compatibility can be introduced into diploid germplasm. However, the majority of diploid potato clones (*Solanum* spp.) possess gametophytic self-incompatibility that is primarily controlled by a single multiallelic locus called the *S*-locus which is composed of tightly linked genes, *S-RNase* (*S*-locus RNase) and multiple *SLFs* (*S*-locus F-box proteins), which are expressed in the style and pollen, respectively. Using *S-RNase* genes known to function in the Solanaceae gametophytic SI mechanism, we identified *S-RNase* alleles with flower-specific expression in two diploid self-incompatible potato lines using genome resequencing data. Consistent with the location of the *S*-locus in potato, we genetically mapped the *S-RNase* gene using a segregating population to a region of low recombination within the pericentromere of chromosome 1. To generate self-compatible diploid potato lines, a dual single-guide RNA (sgRNA) strategy was used to target conserved exonic regions of the *S-RNase* gene and generate targeted knockouts (KOs) using a Clustered Regularly Interspaced Short Palindromic Repeats/CRISPR-associated protein 9 (Cas9) approach. Self-compatibility was achieved in nine *S-RNase* KO T_0_ lines which contained bi-allelic and homozygous deletions/insertions in both genotypes, transmitting self compatibility to T_1_ progeny. This study demonstrates an efficient approach to achieve stable, consistent self-compatibility through *S-RNase* KO for use in diploid potato breeding approaches.

## Introduction

Cultivated potato (*Solanum tuberosum* L.) is the third most important food crop after rice and wheat (Devaux et al., 2014) and plays an essential role in human nutrition as a primary source of carbohydrates. Although global production of potato totaled 376 million tons in 2016 (FAOSTAT, 2019), potatoes face high production losses due to biotic and abiotic stresses that will increase with global warming (Raymundo et al., 2017). While improvement of cultivated potatoes (2*n* = 4*x* = 48) relies on the discovery and introgression of genes from wild species for traits such as disease resistance, the polyploid nature of cultivated tetraploid potato hampers the fixation of desirable alleles in new cultivars. For example, the introgression of critical dominant alleles such as the potato virus Y (PVY) disease-resistance gene in a triplex or quadruplex allelic configuration can take up to 15 years (Mendoza et al., 1996). Re-inventing potato as a diploid inbred/F1 hybrid variety (2*n* = 2*x* = 24) would allow the application of efficient breeding methods (Jansky et al., 2016) as inbred potatoes would accelerate the generation of new varieties with favorable allelic combinations targeting yield, tuber quality, and resistance traits. A significant barrier to this approach is the occurrence of gametophytic self-incompatibility (SI) in a majority of the diploid potato germplasm, thereby preventing the ability to generate diploid homozygous lines.

In diploid potato, the gametophytic SI system is controlled by a single multiallelic locus called the *S*-locus (Porcher and Lande, 2005). This locus is composed of tightly linked genes, *S-RNase* (*S*-locus RNase) and *SLFs* (*S*-locus F-box) genes known also as *S*-haplotype-specific F-box brothers (*SFBB*), expressed in the style and pollen, respectively (Takayama and Isogai, 2005; Sassa et al., 2007; Kubo et al., 2010; Bush and Moore, 2012). The S-RNase protein produces cytotoxic effects that inhibit the elongation of self-pollen tubes via degradation of RNA from the pollen whereas *SLF* functions as a component of a detoxification complex that mediates ubiquitination of non-self S-RNase proteins leading to degradation via the proteasome pathway (Sijacic et al., 2004; Kubo et al., 2015). Hence, when self-pollination occurs in self-incompatible individuals, the *SLFs* genes do not recognize their native *S-RNase* and consequently, pollen tube growth in the style is inhibited due to the ribonuclease activity of the *S-RNase* (Hua et al., 2008).

In an effort to develop diploid self-compatible (SC) potato lines, the inbred line M6 was generated from the wild tuber-bearing species, *Solanum chacoense* (Jansky et al., 2014). In M6, a dominant allele of the *S*-locus inhibitor (*Sli*) inactivates the gametophytic SI system (Hosaka and Hanneman, 1998) leading to self-compatibility. However, introgression of *Sli* into other germplasm is time-consuming and could lead to linkage drag and fixation of undesirable traits such as high tuber glycoalkaloid content from the donor *S. chacoense*. An alternative strategy to *Sli* introgression is the use of genome editing to accelerate the generation of SC diploid lines by targeting genes involved in SI.

Genome editing by Clustered Regularly Interspaced Short Palindromic Repeat (CRISPR)-associated protein 9 (Cas9) system has been widely used to generate gene knockouts (KOs) of candidate genes related to agronomic traits in important crops (Jaganathan et al., 2018). Cas9 induces double-strand breaks (DSBs) in the DNA at the target site, triggering the response of endogenous cell repair mechanisms. One of the cellular mechanisms to repair DSBs is non-homologous end joining (NHEJ), which can generate insertions and deletions in the coding region resulting in a KO of gene function (Bortesi and Fischer, 2015; Pellagatti et al., 2015). The target-DNA recognition is mediated by a single guide RNA (sgRNA) bearing a 20 bp target-site complementary to the region adjacent to a protospacer-adjacent motif (PAM), 5′-NGG-3′, resulting in the generation of a DSB (Doudna and Charpentier, 2014; Sternberg et al., 2014; Pellagatti et al., 2015) which can be leveraged to generate DSB of target genes.

Previous studies in tomato wild relatives demonstrated that missense mutations and gene loss prevent *S-RNase* ribonuclease activity in *S. peruvianum* and *S. pennellii*, leading to self-compatibility (Kowyama et al., 1994; Royo et al., 1994;Covey et al., 2010; Li and Chetelat, 2015). Considering that *S-RNase* is the gametophytic SI component directly implicated in degradation of RNA in self-pollen tubes, inhibiting the *S-RNase* function is a straightforward strategy to confer self compatibility in potato. In an effort to contribute to the development of diploid inbred potato lines, we generated SC diploid lines by targeted mutagenesis of *S-RNase* using CRISPR-Cas9, obtaining stable self-compatibility in T_0_ and T_1_ generations. Contemporaneous with the writing of this manuscript, Ye et al. (2018) published their findings using a similar approach. However, this study provides further insight into SI in diploid potatoes, reporting three new *S-RNase* alleles, their localization within a low recombination pericentromeric region consistent with the location of the *S*-locus, generation of stable SC KO lines, and documentation of plasticity in the phenotype of SI in two diploid lines.

## Materials and Methods

### Plant Material

After an initial test of self-compatibility with more than 50 self-pollinations, the SI diploid potato lines (2*n* = 2*x* = 24) DRH-195 and DRH-310 F_1_ lines were generated from a cross between *S. tuberosum* Gp. Phureja DM 1-3 516 R44 (DM) and *S. tuberosum* Gp Tuberosum RH 89-039-16 (RH) at Virginia Tech and used in this study. Plants were maintained *in vitro*, propagated on Murashige and Skoog (MS) medium (MS basal salts plus vitamins, 3% sucrose, 0.7% plant agar, pH 5.8) (Murashige and Skoog, 1962) and cultured in growth chambers with 16-h-light/8-h-dark photoperiod at 22°C and average light intensity of 200 μmoles m^-2^ s^-1^.

### Allelic Identification, Annotation, and Phylogenetic Analysis of *S-RNase*

TBLASTN [basic local alignment search tool (BLAST)] searches were performed using reported S-RNase protein sequences (Table 1) from the Solanaceae family against the DM v4.04 assembly (Hardigan et al., 2016) using BLAST v2.2.31 (Altschul et al., 1990) with default parameters. A candidate *S-RNase* gene was selected using the top blast hits. Expression abundances across a range of developmental stages, tissues, and organs were determined using available gene expression atlases for DM and RH (The Potato Genome Sequencing Consortium, 2011). To identify *S-RNase* allelic variants in the diploid potato clones, genomic and complementary DNA sequence data from DRH-195 and DRH-310 leaf and tuber tissues were retrieved from the Sequence Read Archive (SRA) of the National Center for Biotechnology Information (Supplementary Table S1) and aligned to the DM v4.04 assembly using BWA-MEM (Li, 2013). Duplicate reads were removed using Picard Tools v1.113^1^ and consensus sequences were obtained using the *mpileup* utility from Samtools v1.2 (Li et al., 2009) with the consensus option from bcftools v1.2 (Li, 2011).

**Table 1:** *S-RNase* sequences from seven Solanaceae species used in this study.

Gene/protein^†^	Accession^‡^	Species
Ribonuclease S-2	Q01796	*Solanum tuberosum*
RNase	CAA05306	*Nicotiana sylvestris*
S-RNase	BAC00940	*Solanum neorickii*
S1-RNase	BAC00934	*Solanum chilense*
S11	AAA50306	*Solanum chacoense*
S2 self-incompatibility ribonuclease precursor	AAG21384	*Petunia integrifolia* subsp. *inflata*
Sx-protein	AAA33729	*Petunia* x *hybrida*


*^†^Gene or protein name, ^‡^National Center for Biotechnology Information (NCBI) accession identifier.*

A primer set was designed to amplify the predicted ORF of the *S-RNase* gene in DRH-195 and DRH-310 using the detected *S-RNase* variants (Supplementary Table S2). S-RNase amino acid sequences reported in Table 1, including the alleles reported by Ye et al. (2018), along with the deduced amino acid sequences from the S-RNase variants identified in this study were aligned using Clustal Omega (Sievers et al., 2011). A phylogenetic tree was constructed using the Neighbor joining method with 1000 bootstrap replicates in *MEGA* version 7.0 (Kumar et al., 2016). Amino acid similarities percentages were calculated using BioEdit (Hall, 1999).

### *S-RNase* Linkage Mapping

The previously reported diploid DRH mapping population was used to genetically map the *S-RNase* gene (Manrique-Carpintero et al., 2015). DNA was isolated from DRH-195 and DRH-310 young leaves using the DNeasy Plant Mini Kit (Qiagen, Hilden, Germany), and used for PCR with a Q5^®^ High-Fidelity DNA Polymerase (New England Biolabs, Ipswich, MA, United States) with the following thermocycler conditions: one cycle of initial denaturation for 4 min at 94°C, followed by 34 cycles for 15 s at 94°C, 45 s at 56°C and 1 min at 72°C and a final extension of 5 min at 72°C. *S-RNase* amplicons were gel-purified using the QIAquick PCR Purification Kit (Qiagen, Hilden, Germany) and cloned into the Zero Blunt TOPO PCR Cloning vector (Thermo Fisher, Carlsbad, CA, United States). Ten colonies for each line were sequenced by the Sanger method and aligned using Clustal Omega (Sievers et al., 2011). DM and RH allelic sequences were confirmed and used to design *S-RNase* RH-allele specific primers (Supplementary Table S2). These primers were screened across 80 individuals of the DRH mapping population. The genotype from the presence/absence of an RH allele was coded as nnxnp and used for mapping in JoinMap4.1 with the same parameters as previously reported by Manrique-Carpintero et al. (2015).

### sgRNA Identification, Assembly, and Validation

A double sgRNA construct targeting predicted conserved regions from the first (sgRNA 1) and second (sgRNA 2) *S-RNase* exons were designed using CRISPR RGEN tools (Supplementary Table S2, Park et al., 2015). A gene KO construct containing the sgRNA combination (sgRNA 1–2) was assembled in the pHSE40 vector containing the CRISPR-Cas9 cassette as described by Xing et al. (2014) and transferred into *Agrobacterium tumefaciens* strain GV3101 pMP90 (Koncz et al., 1994) by electroporation.

### *Agrobacterium*-Mediated Transformation

*Agrobacterium*-mediated transformation was performed using leaf segments from 4-week-old tissue culture plants of DRH 195 and DRH 310 as described by Li et al. (1999). Briefly, explants were pre-cultured on a step I media (MS salts, 3% sucrose, 5 g/l phytoagar, 1 mg/l thiamine-HCl, 0. 8 mg/l zeatin-riboside and 2 mg/l 2,4-D) for 4 days and inoculated with *Agrobacterium*. After 3 days, explants were rinsed with sterile distilled water containing 250 mg/l cefotaxime and 200 mg/l carbenicillin and placed onto step II media (MS salts, 3% sucrose, 5 g/l phytoagar, 1 mg/l thiamine-HCl, 0.8 mg/l zeatin- riboside, 2 mg/l gibberellic acid, 20 mg/l hygromycin and 150 mg/l ticarcillin disodium and clavulanate potassium). Explants were transferred to fresh step II media every week. After approximately 30 days, transformation events (T_0_ lines) were selected from step II media and transferred to root induction media containing MS medium supplemented with antibiotics for selection as described above.

### Molecular Characterization of KO Lines

DNA from T_0_ plants was isolated as described above. PCR was carried out using the GoTaq DNA polymerase (Promega, Fitchburg, WI, United States) with the following thermocycler conditions: one cycle of initial denaturation for 4 min at 94°C, followed by 34 cycles for 15 s at 94°C, 45 s at 56°C and 1 min at 72°C and a final extension of 5 min at 72°C. Amplicons were visualized on 1% (w/v) agarose gels. Allelic mutations of positive transformation events were identified by insertion/deletion presence. Selected transformation events were amplified with the Q5 High-Fidelity DNA Polymerase (New England Biolabs, Ipswich, MA, United States). Then, purified PCR products were cloned into the Zero Blunt TOPO PCR Cloning vector (Thermo Fisher, Carlsbad, CA, United States), and transformed into DH5α competent cells (Thermo Fisher, Carlsbad, CA, United States). Colonies carrying the alleles from each event were Sanger sequenced.

### Assessment of Self-Compatibility

One month old *in vitro* plants were planted in one gallon plastic pots with a peat and perlite growth medium mixture (Bacto professional planting mix) and placed into a greenhouse with a light intensity of 250 μmoles m^-2^ s^-1^, 16/8-h light/dark photoperiod and a temperature of 25°C. Plants were fertilized with Peters Professional^®^ 20: 20: 20 fertilizer (The Scotts, Co., Marysville, OH, United States) at a rate of 500 mg/l twice a week. Around 50 flowers per plants were hand self-pollinated to test for self compatibility. Pollen staining with acetocarmine-glycerol (Ordoñez, 2014b) and cross-pollination were also done to test male and female viability, respectively. T_0_ fruits were harvested 3–4 weeks after self-pollination and kept at room temperature for 2 weeks. Extracted T_1_ seeds were sterilized and subjected to overnight treatment with 1500 ppm of gibberellic acid then allowed to germinate. T_1_ seedlings were transferred to greenhouse and self-pollinated as described above. Additionally, chloroplast counting of guard cells was performed according to Ordoñez (2014a) to discard possible chromosome doubling in each selected *S-RNase* KO line.

### *S-RNase* Expression Analysis

Twenty-five flowers from wild-type (WT) and DRH-195/310-derived T_0_ KO lines (DRH-195.158 and DRH-310.21) were self-pollinated at anthesis. Pollinated pistils were excised 24-hour post pollination (hpp) and preserved in -80°C until use. Total RNA was isolated using the RNeasy Plant Mini Kit (Qiagen, Hilden, Germany) and DNase treated using the TURBO DNA-free kit (Thermo Fisher, Carlsbad, CA, United States) following manufacturer’s instructions. RNA was quantified using a NanoDrop spectrophotometer (Thermo Fisher Scientific, Grand Island, NY, United States) and a reverse-transcription polymerase chain reaction (RT-PCR) was carried-out with 1 μg of total RNA using the Super-Script One-Step RT-PCR kit (Thermo Fisher Scientific, Carlsbad, CA, United States). Primers designed to amplify the *S-RNase* ORF and the elongation-factor one alpha (*EF1α*) housekeeping internal control were used for the RT-PCR reaction (Supplementary Table S2).

## Results

### Identification of the *S-RNase* Gene in Potato

**FIGURE 1:**
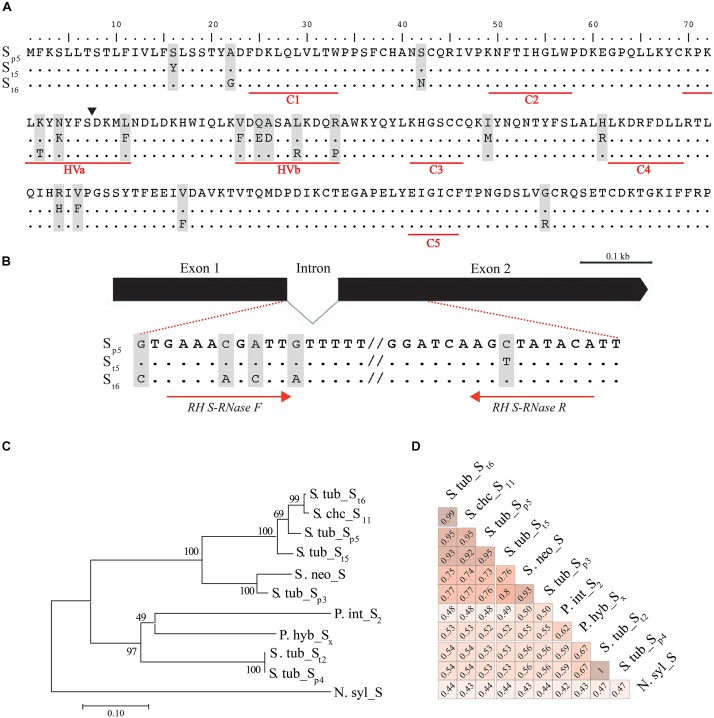
*S-RNase* gene structure and allelic variants in diploid potato and related species. **(A)** S-RNase predicted amino-acid sequence alignment of the DM (S_p5_) and RH (S_t5_ and S_t6_) alleles. Underlined regions in red represent the typical five conserved regions (C1 to C5) and two hypervariable regions (HVa and HVb) of the *S-RNase* gene family. Exon/intron boundary is indicated with a filled triangle within the HVa region **(B)** *S-RNase* gene structure. The *S-RNase* open reading frame is composed of two exons separated by one small intron. Zoomed-in regions are shown within dotted lines indicating the intronic and exonic regions used for RH-specific primer design within the reported *S-RNase* alleles. **(C)** Phylogenetic tree constructed using the Neighbor Joining method based in the proportion of S-RNase amino acid differences. *S-RNase* from *Nicotiana sylvestris* was used as out-group. Numbers above each branch represent bootstrapping percentages from1000 replications. **(D)** Pairwise amino acid similarity of S-RNase in *Solanum* species and detected S-RNase alleles. *S-RNase* alleles are represented first by a species name abbreviation followed by S and the allele number for Tuberosum (t) or Phureja (p) group in *Solanum tuberosum*. For other species, a similar pattern is used, and allele numbers or letters (i.e., Sx for *P. hybrida*) are added if reported. S. tub: *S. tuberosum*, S. chc: *S. chacoense*, S. neo: *S. neorickii*, P. int: *P.* *integrifolia*, P. hyb: *P. hybrida*, N. syl: *N. sylvestris.*

For this study, we used available genomic and gene expression data from the sequenced doubled monoploid DM and the SC heterozygous breeding line RH (The Potato Genome Sequencing Consortium, 2011). To identify the *S-RNase* gene, the DM genome sequence was selected as the reference genome and utilized in sequence similarity searches using seven Solanaceae *S-RNase* genes (Table 1). Candidate genomic regions encoding the *S-RNase* gene were identified and located within 3,948,850–3,949,581 bp of the unanchored scaffold PGSC0003DMB000000091 and the annotated DM *S-RNase* allele, PGSC0003DMG400026738, which encodes a 738 bp open reading frame and a 216 amino acids (aa) predicted protein composed of five conserved and two hypervariable regions, characteristic of *S-RNase*s (Figure 1A; Ioerger et al., 1991). The detected DM and RH *S-RNase* alleles resemble class III *S-RNases* (Figure 1B) and are comprised of two exons and one small intron, which is located at position five of the 11 recognized intron positions for this gene family (Igic and Kohn, 2001; Ramanauskas and Igić, 2017).

We found that the DM *S-RNase* (referred hereafter as *S. tub*_S_p5_ from *S. tuberosum S-RNase* allele five of *S. tuberosum* Group Phureja) is highly expressed in mature flowers [245.5 Fragments Per Kilobase of exon model per Million mapped reads (FPKM)] compared with no expression in leaves or tubers in DM. High *S-RNase* expression levels were also detected in carpels (4342.7 FKPM) consistent with its role in preventing self compatibility (Kao and Tsukamoto, 2004). More limited gene expression data is available for RH (The Potato Genome Sequencing Consortium, 2011) and consistent with the expression of *S-RNase* in potato, it is expressed in flowers (167.04 FPKM) and not tubers or leaves. Together with the functional annotation, these results suggest we have identified the *S-RNase* gene involved in SI.

### Identification of Allelic Variants of *S-RNase* in Diploid Potato Lines

The DRH-195 and DRH-310 F_1_ diploid self-incompatible lines derived from a cross between DM and RH were used to identify *S-RNase* allelic variants. Using whole genome resequencing data for these two lines, the DM and RH *S-RNase* alleles were identified in DRH-195 and DRH-310 and validated using Sanger sequencing. As previously observed for DM, the *S. tub*_S_p5_ predicted 216 amino acid sequence was detected in both lines, beside one of the two RH *S-RNase* alleles in each line, hereafter referred as *S. tub*_S_t5_ and *S. tub*_S_t6_ (*S. tuberosum S-RNase* alleles five and six of *S. tuberosum* Gp Tuberosum, respectively, Figure 1A).

A phylogenetic tree was constructed using available S-RNase amino acid sequences from *Solanum* species and the allelic variants identified in this study (Figure 1C). Two main clades with high confidence bootstrap values were observed. The *S. tub*_S_p5_, *S. tub*_S_t5_, and *S. tub*_S_t6_ alleles and the *S. chacoense* (*S. chc*_S_11_) allele exhibited highest percentage of similarity relative to the other species (Figure 1D and Supplementary Table S3), whereas the *Petunia* (*P. int*_S_2_ and *P. hyb*_S_x_) and *S. tuberosum* (*S. tub*_S_t2_) S-RNases clustered in two separate subgroups. Two *S. tuberosum* Gp Phureja alleles, *S. tub*_S_p3_ and *S. tub*_S_p4_, were located in separate clades (Figure 1C). Overall, the amino acid sequence identity between S-RNase alleles ranged from 42 to 100%, showing 92% similarity between the DM and RH S-RNase alleles and 93% between the two RH alleles (Figure 1D). Notably, the *S. tub*_S_p4_ allele had 100% similarity with a previously reported S-RNase from *S. tub_*S_t2_ (NCBI accession: Q01796), presumably representing the same allele. Moreover, *S. tub*_S_p5_, *S. tub*_S_t5_, and *S. tub*_S_t6_ alleles had similar amino acid identity (53–54%) to the reported *S. tub*_S_p3_ allele when compared with *S. tub*_S_p4_ (Ye et al., 2018).

### *S-RNase* Is Located Within a Pericentromeric Region of Chromosome 1 in Potato

**FIGURE 2:**
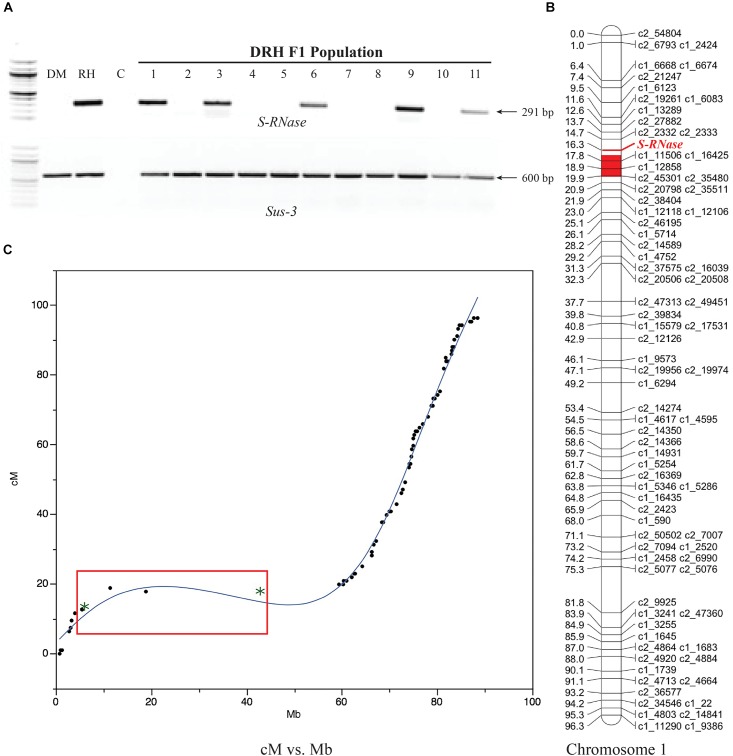
*S-RNase* gene mapping in diploid potato. **(A)** RH *S-RNase* (*S. tub*_S_t6_) allelic screening on the DRH F1 population using the *S-RNase* and housekeeping *Sucrose synthase 3* gene (*Sus-3*) primers. DM and RH parental lines are shown in the first two lanes followed by the negative control (C), and the RH-*S-RNase* segregation pattern of 11 F1-derived lines. **(B)** The *S-RNase* gene mapped to 16.3 cM on the short arm near the centromeric region of chromosome 1 (red). **(C)** Marey map of physical (Mb) versus genetic (cM) distances from chromosome I showing the *S-RNase* gene within a low-recombination region (red box). Asterisks within the red box represent SNPs spanning the region between to 6.1 and 18.9 Mb in the potato physical map (solcap_snp_c2_27882 and solcap_snp_c1_16425 markers, respectively).

Using the segregating DRH population, linkage mapping indicated that the *S-RNase S. tub*_S_t6_ allele mapped to the pericentromeric region of chromosome 1 spanning a region between 13.7 and 17.8 cM (solcap_snp_c2_27882 and solcap_snp_c1_16425 markers, respectively), corresponding to 6.1 and 18.9 Mb of chromosome 1 in the physical map (Figure 2). These results are consistent with the region corresponding to the map location of the *S*-locus in potato (Gebhardt et al., 1991).

### Targeted Mutagenesis of *S-RNase* in Diploid Potatoes Using CRISPR/Cas9 Results in Self-Compatibility

**FIGURE 3:**
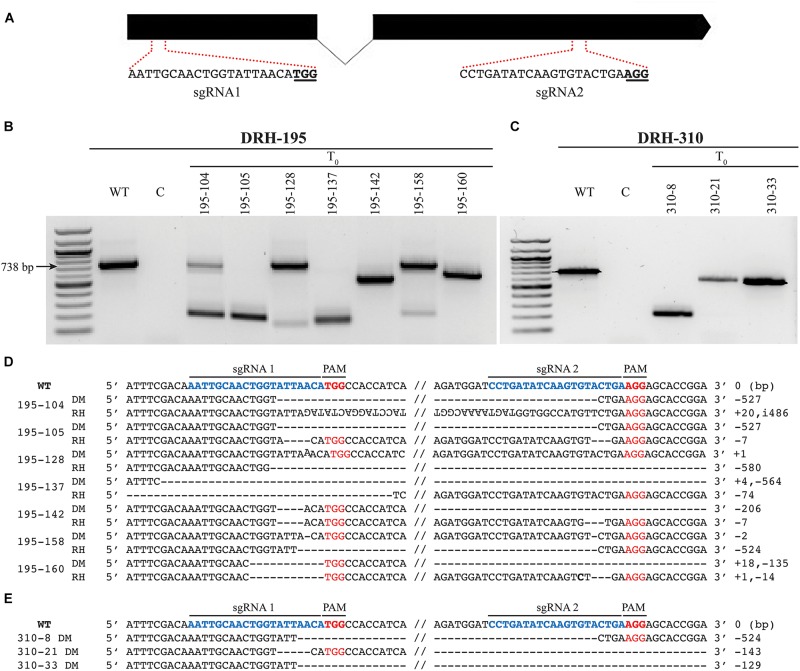
CRISPR/Cas9 mediated mutagenesis in two self incompatible diploid potato lines. **(A)** Single-guide RNAs (sgRNAs) designed to target the *S-RNase* exon1 (sgRNA 1) and exon 2 (sgRNA 2). Zoomed-in regions within the dotted lines show sgRNA and PAM sequences. **(B)** Seven and **(C)** three bi-allelic *S-RNase* knockouts (KOs) DRH-195 and DRH-310 T_0_ lines, respectively, detected by insertion/deletion polymorphisms compared to wild-type (WT) and negative controls (C). All DRH-195 KOs exhibit polymorphic deletions and DRH-310 monomorphic deletions (lines 195–105, 195–137, 195–142, and 195–160 presented a faint mutated or WT-like bands that is not observed in the figure). **(D)** Different mutation types detected by amplicon sequencing in selected KOs for DRH-195, and **(E)** DRH-310 T_0_ lines respectively. Wild-type *S-RNase* exhibiting selected sgRNAs (sgRNA1 and sgRNA2) and PAM sequences are shown at the top of each alignment. Different types of mutations including deletions (–), insertions (+), and inversions (i) detected in DM (DM) and RH (RH) *S-RNase* alleles of each T_0_ KO DRH-derived lines shown as ‘195-’ or ‘310-.’

A dual gRNA strategy (sgRNA 1 and sgRNA 2) was used to efficiently generate *S-RNase* KOs and disrupt the *S-RNase* function in DRH-195 and DRH-310 (Figure 3A). Multiple T_0_ plants were recovered for each line due to a 98% regeneration and transformation efficiency for DRH-195 and 93% for DRH-310 (Table 2). Based upon PCR analysis using primers to the *S-RNase* and gel detection of insertion/deletion polymorphisms, biallelic *S-RNase* mutations were recovered in both the DRH-195 and DRH-310-derived T_0_ lines (Figure 3B,C). Specifically, seven *S-RNase* KOs exhibiting polymorphic deletions with up to 580 bp were detected for DRH-195-derived T_0_ lines. In contrast, for DRH-310, only three *S-RNase* KOs were detected with up to 524 bp monomorphic deletions. To further characterize the CRISPR-targeted regions in selected T_0_ lines, both T_0_ KOs and WT-like *S-RNase* amplicons were sequenced (Figure 3D,E). A distinct nucleotide deletion was detected in each KO line, ranging from small bi-allelic deletions (1 bp) to large homozygous deletions (527 bp) in both *S-RNase* alleles of each DRH-derived T_0_ lines. Insertions (1 to 18 bp) and inversions (486 bp) were also observed in a bi-allelic configuration. Similarly, besides the described mutation types, chimeric mutations were detected in T_0_ lines, which has been reported in other species subjected to CRISPR-mediated mutagenesis, potentially due to late embryogenesis editing (Gomez et al., 2018).

**Table 2:** DRH-195 and DRH-310-derived T_0_ and indel-based selected *S-RNase* KO lines with bi-allelic mutations.

Line	Number of explants	T_0_ lines	Transformation efficiency (%)*	Mutant deletion polymorphism type	
				*Single*	*Double*
DRH-195	186	162	98		7
DRH-310	276	78	93	3	


*^∗^Calculated as the percentage of T_0_ lines with Cas9 integration.*

During the regeneration process the high rate of cell division can induce chromosome doubling. To test whether the selected T_0_ KOs lines underwent spontaneous chromosome doubling, chloroplasts were counted in stomatal guard cells. One out of 10 *S-RNase* KO lines (DRH-195.104) revealed chromosome doubling which has also been observed in a related *S-RNase* KO approach (Ye et al., 2018). This phenomenon, known as endopolyploidization, is frequently observed in potatoes subjected to regeneration processes, in which structural cell and chromosome rearrangements at mitosis results in increased chromosome numbers (Karp et al., 1984; Owen et al., 1988). The tetraploid KO line was not considered for further analysis.

**FIGURE 4:**
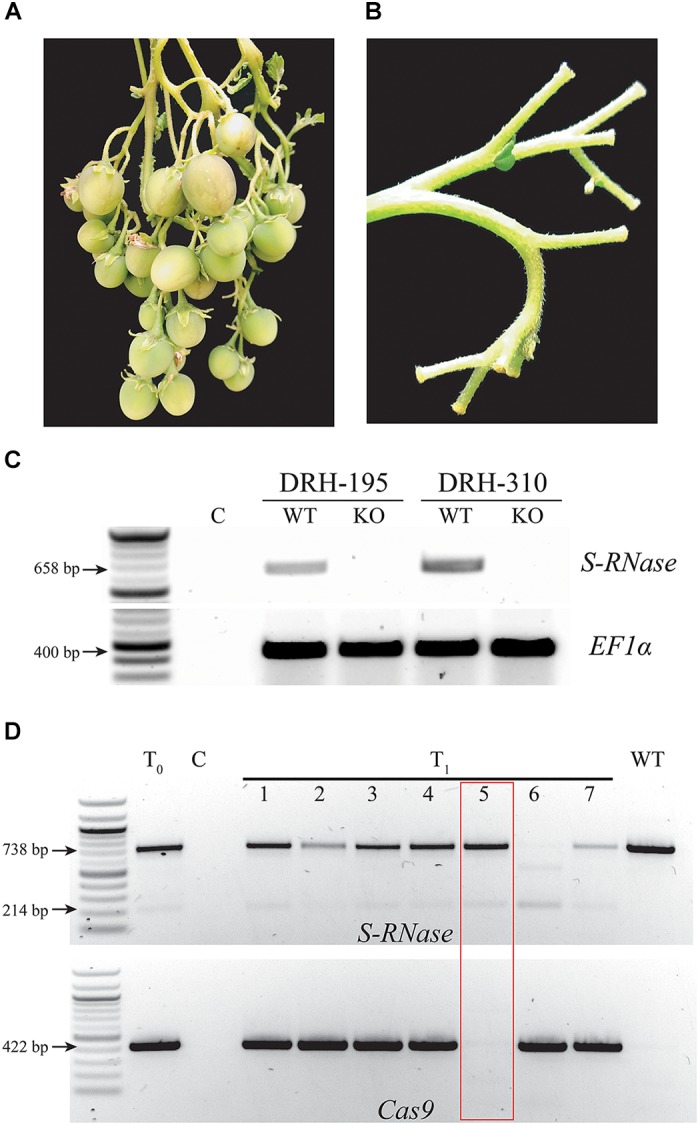
*S-RNase* expression and KO phenotype in self incompatible diploid potato lines. **(A)** Fruits obtained after 5 weeks of self-pollination in an *S-RNase* DRH-195-derived T_0_ mutant line. **(B)** Dropped flowers after self-pollination in the WT DRH-195. **(C)** Semi-quantitative reverse transcription PCR (RT-PCR) in DRH-195 and DRH-310 self-pollinated WT and KO T_0_ lines (DRH-195.158 and DRH-310-21, respectively). RNA was isolated from pistils 24 h after self-pollination revealing *S-RNas*e expression in WT but not in KO lines as compared with the housekeeping gene control (EF1α). **(D)** T_1_ plants derived from the DRH-195.158 T_0_ line were screened with the *S-RNase* primers. WT-like bands with 1 bp deletion on each target site (causing frameshift leading to a premature stop codon, Supplementary Figure S1) are observed in T_0_ and T_1_ lines. Cas9 gene did not transmit to T_1_ line 5 (lane 5). A previously undetected band observed in lane 6 is potentially the result of transgenerational CRISPR/Cas9 activity. T_0_: DRH-195.158, C: Negative control. The red box is showing a T_1_ line segregating out Cas9 while maintaining the *S-RNase* KO.

**FIGURE 5:**
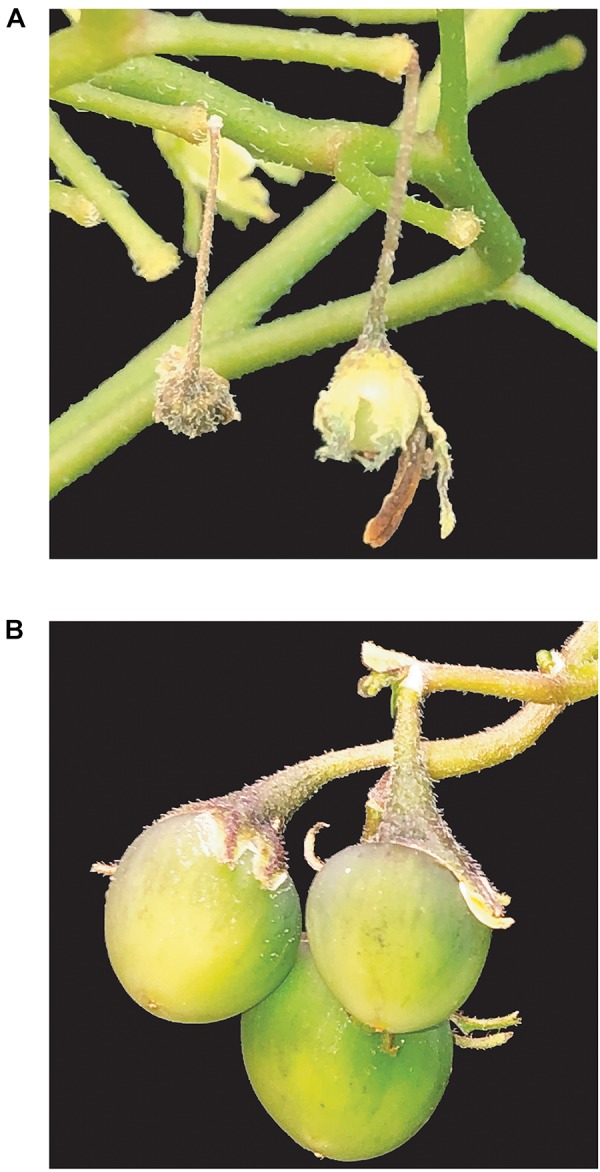
Fruit formation in WT and T_1_ *S-RNase* KO diploid potatoes lines. **(A)**. Fruit setting arrest 2 weeks after self-pollination in the WT DRH-310. **(B)** Fruits obtained after 4 weeks of self-pollination in an *S-RNase* DRH-195-derived T_1_ mutant line.

To confirm the *S-RNase* mutant phenotype, T_0_ KO lines were self-pollinated in two separate replications under greenhouse conditions. In both replications, all T_0_ KO lines set fruit (Figure 4A and Supplementary Tables S4, S5). In one of the replications, both WT non-transformed lines (DRH-195 and DRH-310) also exhibited a limited number of specific self-pollination events with fruit set that either had complete development (DRH-195, Supplementary Table S4) or arrest of fruit set 2 weeks after self-pollination (DRH-310, Figure 5A). However, after 1 week, new self-pollinations of WT lines did not set fruit (Figure 4B) suggesting plasticity of self-compatibility, a phenomenon observed previously in *Solanum* (Saba-El-Leil et al., 1994; Mena-Ali and Stephenson, 2007). This plasticity however, represents an unreliable source of self-compatibility as was evident in the ratio of fruit set per pollination observed, with self-pollination success in *S-RNase* KO lines being an order of magnitude higher than the WT in both DRH-195 and DRH-310 KO lines (Supplementary Tables S4, S5). To further investigate if this result was associated with the suppression of *S-RNase* expression, a semi-quantitative RT-PCR was performed. As shown in Figure 4C, *S-RNase* transcripts were detected in both WT lines 24 hpp yet no expression was detected in T_0_ KOs, confirming *S-RNase* expression in WT but not mutant lines.

Viable T_1_ seeds were obtained for each *S-RNase* T_0_ KO line. Self-compatibility was confirmed in T_1_ lines after self-pollination demostrating the inheritance and stablity of the *S-RNase* KO phenotype (Figure 5B). Cas9 inheritance in the T_1_ lines exhibited a segration ratio associated with a hemizygous multi-copy integration of Cas9 (4 out of 135), in addition to the segregation of the mutated *S-RNase* alleles (Figure 4D). Likewise, because of the activity of integrated Cas9, a potential transgenerational deletion was observed in a T_1_ line (DRH-195.158.6). These results demonstrate the advantage of using CRISPR/Cas9 to generate Cas9-free edited plants and the potential to transmit stable gene mutations through different generations.

## Discussion

Self-incompatibility has been a limiting factor for inbred/F_1_ hybrid cultivar development in diploid potatoes because efforts involving crossing with wild SC relatives result in many undesirable traits segregating in the progeny. To redirect potato breeding toward an efficient inbred/F_1_ hybrid generation strategy, we exploited the *S-RNase*-based SI system in diploid potatoes and generated KO lines using CRISPR-based genome editing to achieve self-compatibility.

Amino acid sequence variation within S-RNase was observed among *S. tub*_S_p5_, *S. tub*_S_t5_, and *S. tub*_S_t6_ alleles (Figure 1A). Nearly half of these variants were within the hypervariable domains (HVa and HVb) and not in conserved domains (C1-5) consistent with data that show the *S-RNase* variable regions are the determinants for allele specificity in different *Solanum* species (Matton et al., 1997, 1999; Brisolara-Corrêa et al., 2015). Specifically, four amino acids within these variable regions (T74, N76, Y77, and R101) have been reported as the sole factors for allele conversion of the pollen rejection phenotype in *S. chacoense* (Matton et al., 1997). Three of these amino acid changes were present within the *S. tub*_S_p5_, *S. tub*_S_t5_, and *S. tub*_S_t6_ alleles indicating that these variations could be sufficient to confer allele specificity while preserving their catalytic activity which is associated with two of the five conserved domains (Kao and Tsukamoto, 2004).

Ioerger et al. (1990) observed that *S-RNase* inter-specific similarities were higher than intra-specific similarities in Solanaceae, concluding that *S-RNase* divergence pre-dates speciation in this clade. The results observed in our study further confirm this previous observation. A high degree of inter-specific S-RNase amino acid sequence similarity was observed in *Solanum* *S-RNases* (*S. tuberosum* and *S. chacoense*). Conversely, a clear intra-specific separation within the *S. tuberosum S-RNase* alleles (Figure 1C) was also observed, consistent with the hypothesis of a single ancestral origin of *S-RNase* and conservation of specific polymorphisms throughout evolution governing allelic diversity (Ioerger et al., 1991; Dzidzienyo et al., 2016).

The *S-RNase* gene mapped to chromosome 1 within a region of low recombination consistent with the hypothesis to promote outbreeding due to a reduction in recombination events between the *S-RNase* and *SLF* genes (Kubo et al., 2015; Fujii et al., 2016). This chromosome position has also been reported in other Solanaceae members. For instance, *S-RNase* is located on chromosome 1 in *S. lycopersicum* and *S. peruvianum* within highly complex and repetitive genomic regions (Kubo et al., 2015; Fujii et al., 2016). Furthermore, Kubo et al. (2015) mapped an *SLF*, the other component of the *S*-locus, also to chromosome 1 in potato genome within a repeat-rich sub-centromeric region, suggesting that the *S-RNase* location was at the same position since these genes are reported to be closely linked (Sijacic et al., 2004).

All edited T_0_ lines had a frameshift in the coding region close to sgRNA 1, leading to a premature stop codon. The resulting truncated sequence prevented the amplification of the *S-RNase* gene by removing the primer annealing site at the 3′ end (Supplementary Figure S1). Similarly, the consistent mutations generated by the two sgRNAs allowed detection of *S-RNase* size polymorphisms. It should be noted that this strategy was selected for the potential to use PCR for a quick and facile screen for large deletions in T_0_ lines. However, undetected insertions/deletions or inversions could be present in T_0_ lines. For instance, sequencing data revealed a single bp insertion and deletion in the DM *S-RNase* allele of DRH-195.128 and DRH-195.158, respectively, showing a similar amplicon size in agarose gels as WT (Figure 3B,D). These observations indicate that a large number of allelic KOs can be generated given the high transformation efficiency observed in both diploid lines.

The DRH-195.158 T_0_ KO line, which exhibited a single bp deletion at each sgRNA targeting site in the RH *S. tub*_S_p5_ allele (Figure 3D), showed a new *S-RNase* deletion in a T_1_-derived line (Figure 4D). Given the Cas9 mismatching tolerance, this allele possibly underwent a new mutagenesis event, displaying a different mutation pattern in the T_1_ generation. In different plant species, it has been found that editing occurs at a higher frequency across generations, therefore new mutations segregate from WT alleles in heterozygous T_0_ as a result of constitutive expression of Cas9 (Feng et al., 2014; Xu et al., 2015; Wang et al., 2018).

Two independent self-pollination assays were conducted in DRH-195 and DRH-310 WT lines in 2015 and 2018 with a minimal of 50 flowers, demonstrating their SI. However, a third biological replicate in 2018 resulted in fruit formation suggesting plasticity in the strength of SI. Environmental effects along with plant phenology have been associated with unstable SI in angiosperms. For instance, temperature fluctuations, photoperiod, glucose starvation, and humidity significantly reduced SI in *S. peruvianum* after selfing (Webb and Williams, 1988). This process, known as pseudo-self-incompatibility, has also been reported in grasses in which artificial self-pollination techniques can contribute to SI breakdown (Do Canto et al., 2016). Similarly, sporadic fruit set has been observed across Solanaceae species such as *Witheringia solanacea*, *S. carolinense*, *S. peruvianum*, and *N. alata* in which floral age, flowering stage, and delayed floral abscission has been associated with fruit set in SI populations (Stone et al., 2006; Mena-Ali and Stephenson, 2007; Miller and Kostyun, 2011; Liao et al., 2016). This phenomenon has also been observed in species under sporophytic SI, in which floral age reduces the expression of the *S*-locus associated genes in *Brassica oleracea* resulting in SI breakdown (Hadj-Arab et al., 2010). In natural populations of *Campanula rapunculoides*, strong SI has been observed in young flowers. However, self-fruit formation is also evident in old flowers as a consequence of pollen scarcity and low fruit production from prior inflorescences (Stephenson et al., 2000). Therefore, environmental conditions favoring SI breakdown (plant age, plant health, and greenhouse conditions) could lead to fruit set in one of the WT biological replicates in this study.

Unlike pseudo-self-incompatibility, the *S-RNase* KO proved to be both stable and consistent across different replications and generations, presenting a higher ratio of fruit set per pollination when compared with self-fruit WT lines (Supplementary Tables S4, S5). Although the SC phenotype appears to be line dependent, distinctive *S-RNase* KO lines exhibited either high fruit set or seed formation. These results also indicated that genes other than *S-RNase* could be contributing to the strength of the SI response in both WT and *S-RNase* KOs. In fact, besides the *S-RNase* gene, other SI modifier loci can modulate the pollen rejection response in several *Solanum* species (McClure et al., 1999; O’Brien et al., 2002; Goldraij et al., 2006). For instance, *S-RNase*-independent stylar factors such as eEF1A or High Top-Band (HT-B) proteins, can directly or indirectly interact with *S-RNase* contributing to the SI response (Goldraij et al., 2006; Soulard et al., 2014). Similarly, unintended somaclonal variation and chromosomal rearrangements associated with the potato regeneration processes and Cas9 activity, respectively, could also contribute to variations in the observed ratio of fruit set per pollination within the *S-RNase* KOs.

This hypothesis is further supported by Peterson et al. (2016) which identified several genomic regions associated with self-fertility in a DRH F_1_ population, located on chromosomes IV, IX, XI, and XII. They also found that a specific Single Nucleotide Polymorphism (SNP) associated with the RH allele, fixed in selfed populations, is likely the primary factor for self-fertility in the DRH F_1_ progeny. Overall, this study demonstrates that *S-RNase* is the primary component for self-pollen rejection in DRH-195 and DRH-310. However, external evidence suggests that besides RH self-fertility mechanisms, *S-RNase*-independent stylar factors and environmental conditions could play a role in spontaneous self-compatibility observed in the WT lines in this study.

In this study, we generated self-compatible potato diploid lines by targeting the *S-RNase* gene using the CRISPR-Cas9 system. We first computationally identified three new *S-RNase* alleles in self incompatible diploid lines (a DM and two RH alleles, each inherited to DRH-195 and DRH-310, respectively) and mapped this gene to chromosome 1 within the peri-centromeric region consistent with the localization of the *S*-locus to a low recombination region on chromosome 1. *S-RNase* KO lines were obtained using a dual sgRNA strategy in which premature stop codons were generated. After self-pollination, fruits were set in selected KO lines in T_0_ and T_1_ lines. Cas9-free KO lines were also identified in T_1_ lines. Our results demonstrated the inheritance and stablity of the *S-RNase* KO phenotype, which can contribute to utilization of SC as a first step for the generation of commercial diploid cultivars.

## Data Availability

Publicly available datasets were analyzed in this study. This data can be found in Supplementary Table S1.

## Author Contributions

FE-R and DD conceived the research idea. FE-R, NM-C, SN, CB, DZ, and DD designed the research experiments, analyzed the data, and wrote the manuscript. FE-R performed laboratory experiments. FE-R and NM-C performed self-pollination assays.

## Conflict of Interest Statement

The authors declare that the research was conducted in the absence of any commercial or financial relationships that could be construed as a potential conflict of interest.

## Footnotes

1 http://broadinstitute.github.io/picard

## Supplementary Material

FIGURE S1 *S-RNase* open reading-frames in T_0_ knockout (KO) lines derived in two SI diploid potato lines. Sequences shown within the dotted lines contain 5′ and 3′ mRNA borders of this gene. Primer sequences designed to amplify the *S-RNase* ORF are shown as well as the sgRNA 1 located in exon 1. Individual dots represent nucleotides within the *S-RNase* gene. All T_0_ KO lines had a frameshift near the sgRNA 1 target region (blue dotted line) creating a premature stop codon (red dotted line). Black dotted line represents in frame *S-RNase* regions and nucleotides. Black dotted dash lines represent missing transcript sequence. *S-RNase* alleles from DM and RH are shown for each KO line.

TABLE S1 Retrieved Illumina reads for genomic assembly of the DRH potato lines and assembly statistics.

TABLE S2 Primers and sgRNAs used in this study.

TABLE S3 Top TBLASTN hits of reported S-RNase proteins in the DM genome assembly.

TABLE S4 Fruit set and seed count upon self-pollination in DRH-195 wild-type and *S-RNase-*derived KO lines.

TABLE S5 Fruit set and seed count upon self-pollination in DRH-310 wild-type and *S-RNase-*derived KO lines.
