# Supplementary figures and images for: Overcoming Self-Incompatibility in Diploid Potato Using CRISPR-Cas9
Source: Front Plant Sci. 2019 Apr 2;10:376. doi: 10.3389/fpls.2019.00376 (PMC6454193; doi:10.3389/fpls.2019.00376)

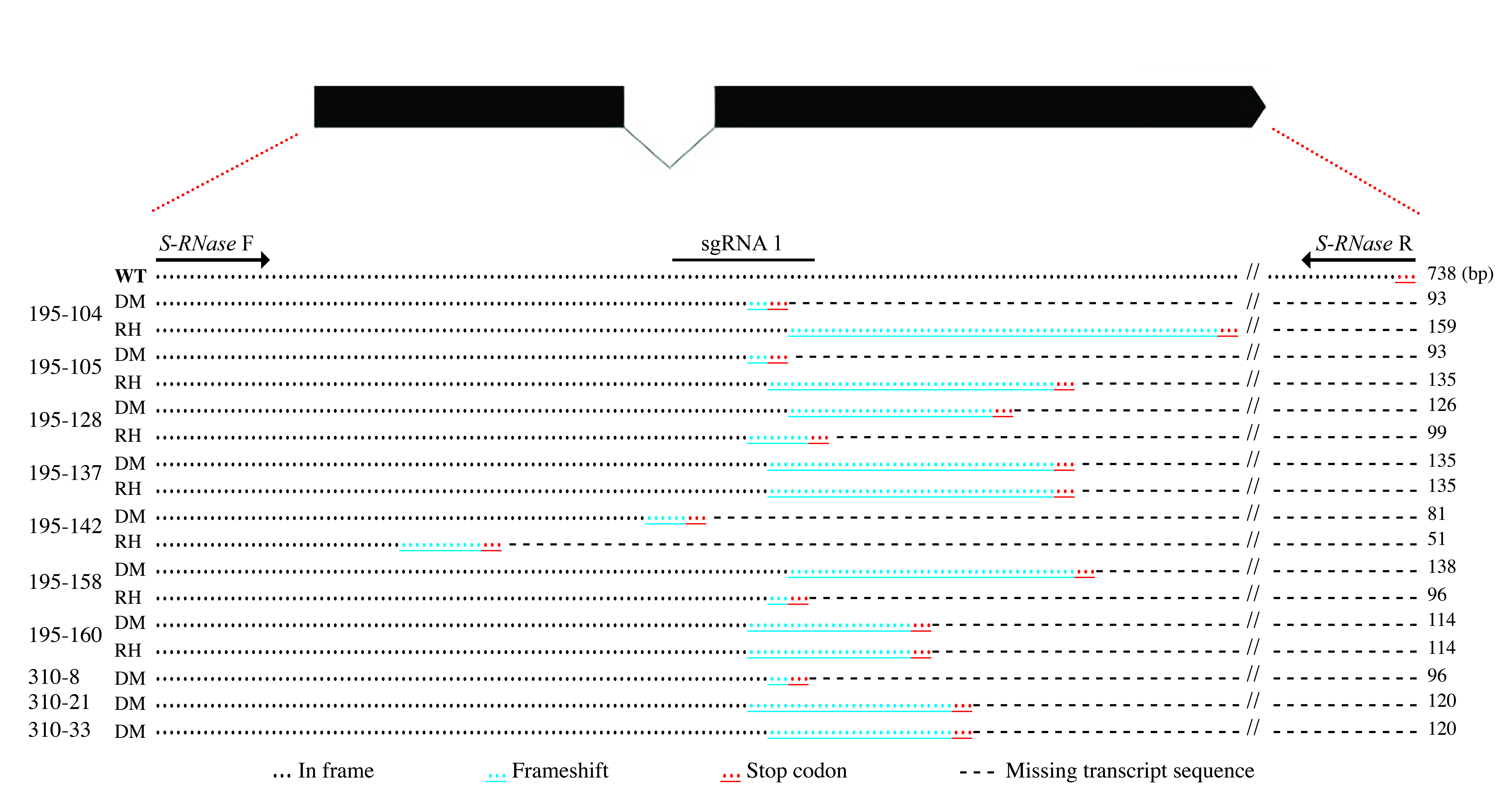

Supplement: FIGURE S1 — S-RNase open reading-frames in T0 knockout (KO) lines derived in two SI diploid potato lines. Sequences shown within the dotted lines contain 5′ and 3′ mRNA borders of this gene. Primer sequences designed to amplify the S-RNase ORF are shown as well as the sgRNA 1 located in exon 1. Individual dots represent nucleotides within the S-RNase gene. All T0 KO lines had a frameshift near the sgRNA 1 target region (blue dotted line) creating a premature stop codon (red dotted line). Black dotted line represents in frame S-RNase regions and nucleotides. Black dotted dash lines represent missing transcript sequence. S-RNase alleles from DM and RH are shown for each KO line. [file Image_1.jpg]
