# Supplementary material for: Overcoming Self-Incompatibility in Diploid Potato Using CRISPR-Cas9
Source: Front Plant Sci. 2019 Apr 2;10:376. doi: 10.3389/fpls.2019.00376 (PMC6454193; doi:10.3389/fpls.2019.00376)
Supplement: TABLE S1 — Retrieved Illumina reads for genomic assembly of the DRH potato lines and assembly statistics. [file Table_1.docx]

**Supplementary tables**

**Supplementary Table 1.** Retrieved Illumina reads for genomic assembly of the DRH potato lines and assembly statistics

| ***Tissue*** | **DRH-195** | | **DRH-310** | |
| --- | --- | --- | --- | --- |
|  | **SRA accession** | **Pair-end Reads** | **SRA accession** | **Pair-end Reads** |
| Mature leaflet | SRR4018191 | 30,799,234 | SRR4018197 | 24,891,913 |
| Leaf | SRR4018147 | 30,423,428 | SRR4018153 | 29,113,282 |
| Tuber | SRR4018170 | 31,800,294 | SRR4018174 | 31,283,414 |
| **Total:** | | 93,022,956 |  | 85,288,609 |

**Supplementary Table 2**. Primers and sgRNAs used in this study.

| **Name** | **Sequence** | **Description** |
| --- | --- | --- |
| S-RNase F | ATGTTTAAATCACTGCTTACATCAAC | *S-RNase* forward primer |
| S-RNase R | TCAGGGACGGAAAAATATTTTCCCTG | *S-RNase* reverse primer |
| S-RNaseRH F | GTTTTGTTTAATTTACTGAAAAGCTTA | RH-specific *S-RNase* forward primer |
| S-RNaseRH R | AAAGATTTCTTCAAATGTATAACT | RH-specific *S-RNase* reverse primer |
| EF1α F | GGTGGTTTTGAAGCTGGTATCTCT | Elongation factor one alpha forward primer |
| EF1α R | CCAGTAGGGCCAAAGGTCACA | Elongation factor one alpha reverse primer |
| sgRNA1 | AATTGCAACTGGTATTAACA**TGG**^†^ | Single-guide RNA 1 targeting exon 1 |
| sgRNA2 | CCTGATATCAAGTGTACTGA**AGG** | Single-guide RNA 2 targeting exon 2 |

^†^ In bold, protospacer adjacent motif (PAM) sequence

**Supplementary Table 3**. Top TBLASTN hits of reported S-RNase proteins in the DM genome assembly.

| **Species** | **Scaffold** | **HSP**^†^ **Number** | **Hit Score** | **E value** | **Length** | **Percent Id** |
| --- | --- | --- | --- | --- | --- | --- |
| *Nicotiana sylvestris* (CAA05306.1) | chr00 | 1 | 192 | 8E-55 | 244 | 41.8 |
| *Petunia integrifolia*  (AAG21384.1) | chr00 | 1 | 159 | 3E-43 | 222 | 44.14 |
| *Petunia x hybrida* (AAA33729.1) | chr00 | 1 | 226 | 2E-66 | 251 | 48.21 |
| *Solanum chacoense* (AAA50306.1) | chr00 | 1 | 415 | 3E-132 | 245 | 84.08 |
| *Solanum chilense* (BAC00934.1) | chr00 | 1 | 313 | 1E-96 | 244 | 63.93 |
| *Solanum neorickii*  (BAC00940.1) | chr00 | 1 | 321 | 2E-99 | 244 | 64.75 |
| *Solanum tuberosum* (Q01796.1) | chr00 | 1 | 204 | 1E-58 | 230 | 49.57 |

^†^ High-scoring Segment Pair

**Supplementary Table 4**. Fruit set and seed count upon self-pollination in DRH-195 wild type and *S-RNase-*derived KO lines

| Line | Self-pollinated flowers^‡^ | Total fruit set | Ratio of fruit set per pollination | Number of cluster and seeds per cluster |
| --- | --- | --- | --- | --- |
| DRH-195 (WT) ^†^ | 130 | 4 | 0.03 | 50 |
| DRH-195.105 | 38 | 29 | 0.76 | 200 |
|  |  |  |  | 60 |
|  |  |  |  | 240 |
| DRH-195.128 | 80 | 9 | 0.11 | 19 |
|  |  |  |  | 7 |
|  |  |  |  | 21 |
|  |  |  |  | 6 |
|  |  |  |  | 10 |
|  |  |  |  | 11 |
| DRH-195.137 | 29 | 14 | 0.48 | 80 |
|  |  |  |  | 24 |
|  |  |  |  | 34 |
|  |  |  |  | 50 |
| DRH-195.142 | 125 | 36 | 0.29 | 20 |
|  |  |  |  | 30 |
|  |  |  |  | 40 |
|  |  |  |  | 50 |
|  |  |  |  | 250 |
|  |  |  |  | 30 |
| DRH-195.158 | 121 | 46 | 0.38 | 50 |
|  |  |  |  | 100 |
|  |  |  |  | 40 |
|  |  |  |  | 50 |
|  |  |  |  | 38 |
|  |  |  |  | 19 |
|  |  |  |  | 51 |
|  |  |  |  | 14 |
|  |  |  |  | 38 |
|  |  |  |  | 45 |
|  |  |  |  | 39 |
|  |  |  |  | 45 |
|  |  |  |  | 226 |
|  |  |  |  | 50 |
|  |  |  |  | 50 |
| DRH-195.160 | 71 | 23 | 0.32 | 200 |
|  |  |  |  | 29 |
|  |  |  |  | 8 |
|  |  |  |  | 33 |
|  |  |  |  | 11 |
|  |  |  |  | 15 |
|  |  |  |  | 200 |

^†^ Wild type. ^‡^ Total of flowers in two replicates.

**Supplementary Table 5**. Fruit set and seed count upon self-pollination in DRH-310 wild type and *S-RNase-*derived KO lines

| Line | Self-pollinated flowers^‡^ | Total fruit set | Ratio of fruit set per pollination | Number of cluster and seeds per cluster |
| --- | --- | --- | --- | --- |
| DRH-310 (WT) ^†^ | 123 | 0 | 0 | 0 |
| DRH-310.8 | 38 | 5 | 0.13 | 2 |
|  |  |  |  | 3 |
|  |  |  |  | 15 |
| DRH-310.21 | 52 | 16 | 0.31 | 50 |
|  |  |  |  | 50 |
|  |  |  |  | 20 |
|  |  |  |  | 28 |
|  |  |  |  | 50 |
|  |  |  |  | 63 |
| DRH-310.33 | 53 | 15 | 0.28 | 10 |
|  |  |  |  | 19 |
|  |  |  |  | 20 |
|  |  |  |  | 20 |
|  |  |  |  | 18 |
|  |  |  |  | 10 |

^†^ Wild type. ^‡^ Total of flowers in two replicates
